# Health Facility Readiness for Promoting Male Involvement in Family Planning Services in Tanzania: A Qualitative Study on Perspectives of Health Providers in Kibaha District

**DOI:** 10.24248/eahrj.v6i1.675

**Published:** 2022-07

**Authors:** Judith Msovela, Anna Tengia – Kessy, Godfrey M. Mubyazi

**Affiliations:** a National Institute for Medical Research, Dar es Salaam, Tanzania; b Department of Community Health, Muhimbili University of Health and Allied Sciences, Dar es Salaam, Tanzania

## Abstract

**Background:**

The successes and failures of health policies and programs to motivate men to develop interest in positive decision making and actions relating to reproductive and child health services (RCHs) in Tanzania are inadequately documented. Therefore, a study was done to explore the health facility readiness for motivating men to effectively participate in RCHs including Family Planning (FP) in Kibaha District.

**Methods:**

This was a cross-sectional qualitative study undertaken in 2014 and involving in-depth interviews (IDIs) with frontline RCH providers at selected Health Facilities (HFs) and their district coordinator. Data were transcribed verbatim, coded and evaluated thematically through a narrative analysis approach.

**Results:**

All the respondents admitted the role of men in influencing FP decisions at family and other community levels and the need for engaging them in RCHs. They all reported to have continued to note an increasing attitude and activeness of men to attend the RCH facilities available for RCHs along with their partners despite the remaining ones who still show hesitance. Family Planning interventions supported by the District Council Authority and development partners were reported to contribute in increasing the number of males coming for FP and other RCHs. Nevertheless, some shortcomings were experienced, and were reported to include some HFs providing FP services on selected week days which limit the clients who would need them any day/time; some dispensaries lacking adequate lounges or consultation room spaces for accommodating clients arriving in couples or who would be held for receiving health education in group; and the occasional stock-outs of essential FP commodities and other RCHs at some of the HFs.

**Conclusions:**

The study reveals the pleasure frontline RCHs staff had after observing an increasing trend in male involvement in such services and the support given by the government and its allied stakeholders to make this a success. However, the prevailing deficiencies relating to HF infrastructure and FP commodity supplies need to be addressed if a universal health coverage for FP and other RCHs were to be attained as policy and program-wise advocated.

## BACKGROUND

Men in developing countries play an influential role in decision making with regard to health care seeking for Reproductive and Child Health Services (RCHs), including among others, Family Planning (FP), Antenatal Care (ANC), skilled delivery and postnatal care.^1,2^ Male involvement in maternal and reproductive health aspects has generally shown to improve FP, increase access to postpartum services, reduce maternal smoking and depression and reduction in infant mortality risk.^3–6^ Male involvement in reproductive health issues continues to gain attention due to men's role at household/family and other social levels.^7^ In spite of this recognition, the progress so far made towards engaging men in maternity care affairs such as Prevention of Mother to Child Transmission (PMTCT) of the Human Immune-Deficiency Virus (HIV), FP and other RCHs has remained low in most developing countries.^8,9^ There are various factors contributing to this low male engagement and these include; long travel distance to reach a health facility (HF); inconvenient clinic hours; long waiting time at the health facility/clinic; unfriendly attitude of some health service providers; limited space to accommodate couple counselling at the HF/clinic; and financial and time costs involved in accessing the services. These glitches discourage men from participating e?ectively in maternal and other RCH programs.^10,11^ Young men's limited use of sexual and reproductive health services has also been found to be associated with the prevailing traditions and norms leading to certain unfair beliefs and/or practices – ‘stereotypes’ like masculinity (e.g. perceived male status, toughness and anti-femininity). There's vast literature testifying that this experience has prevailed in Africa and numerous other communities outside Africa for a long time.^12^ In those areas/communities, FP programs are previously reported to be targeting mostly women, therefore, disengaging men. Even beyond Africa, studies carried out in developed countries such as the US, reported presence of groups of people of African origin and other racial origins sharing negative perception and therefore maintaining stigma against reproductive health services, especially those requiring male involvement in contraceptive use and attending RCH clinics.^12,13,19^ This calls for urgent need for interventionists (reproductive and child health program officers, including planners, managers/administrators and frontline program implementers) to review their strategies and devise means aimed at promoting males' engagement in RCHs. This can be achieved through; strengthened or enhanced community/public education and sensitisation programs, strategically targeting men, including both adults and adolescent boys and the male youth.^20^

Tanzania has not lagged behind in promoting health policies, planning and intervention programs aimed at increasing male involvement or participation in RCH issues. In recognition of the role of men in influencing women's utilisation or uptake of existing reproductive health services, the ministry of health (MoH) is promoting male involvement as an integral element of the existing strategies for FP and promotion of other RCH programs/interventions. There are clear policy statements and guidelines for that, and most of these require multi-sector and multidisciplinary approaches.^3,7^ Such Policy and programs/arrangements include; the National Road Map Strategic Plan to Accelerate Reduction of Maternal, Newborn and Child Deaths 2008-2015,^21^ the National Adolescent Health and Development Strategy 2018,^22^ National Family Planning Guidelines and Standards,^23^ the National Strategy for Prevention of Mother-to-Child Transmission of HIV, and the Health Sector Strategic Plan IV. These documents highlight among other things, the importance of Universal Health Coverage (UHC) in relation to maternal, reproductive, adolescent and child health services.^24^ Encouragingly, a number of interventions have been officially instituted for the purpose of motivating and promoting men involvement in reproductive health issues at all levels.^25,26^ The encouragement of males/men to utilise some Reproductive Health commodities such as condoms could also be a catalyst for not only prevention of Sexually Transmittable Infections (STIs) like those associated with HIV,^27^ but also prevention of unplanned pregnancies.

In an attempt to find a basis for rationalising the present study, vast literature regarding utilisation of RCH programs in Tanzania was searched and studied. These studies did not report sufficient evidence on the extent to which the existing structures and organisation of health service delivery points support or encourage men to participate in RCHs. Based on these findings, analysis of health facilities readiness to promote male involvement in family planning services in Kibaha District, Tanzania was deemed justifiable and timely.

## METHODS

### Study Design

The study deployed a cross-sectional, mixed method survey study design. Data collection and analysis was conducted between April and June, 2014.

### Study Setting

The study was conducted in Kibaha District, Eastern Tanzania. Kibaha District is geographically close to Dar Es Salaam Region. It forms one of the six administrative districts of Pwani Region.^33^ The region is reported to be among regions with not only low FP usage coverage in Tanzania^34^ but also with a decreasing trend in FP service usage.^35^

The latest National Population Census conducted in 2012 showed the Kibaha District Council to have had a total population of 75,899. Among these, women of reproductive age (15-49 years) were 19,015 whereas men aged 15 years and above were 15,598.^36^ The District had a total of 24 HFs of which one was a public (government) Health Centre (HC); 23 were dispensaries (16 of them being public; 12 faith-based organisations (FBOs); 2 private/for-profit – ‘commercial’; whereas 3 were owned and run by parastatal organizations).

### Study Participants and Sampling Techniques

Since the study sought to create an understanding on the extent to which the existing health service delivery points support or encourage men to participate effectively in FP and other RCH programs in Kibaha District, this information could be obtained from respondents who are dealing with provision of these services directly to male clients, as well as their superiors at health facility and district levels. The study also included Community Health Workers (CHWs) since they also deal with provision of health education and other health related services at community level according to the existing service providers’ supervision and guidance policies from the National Guideline.^37^ Simple Random Sampling Technique was used to identify Kibaha as the district for this study, out of the six districts forming the Pwani Region.

Study participants were identified at different levels, namely; the – ‘health facility’, ‘community’ and ‘district’ levels. While they were purposively sampled to respond as key informants (KIs), they were reached at through a multistage sampling strategy. The selected participants were as follows: at the district level (1 district reproductive and child health coordinator – ‘DRCHCo’); at health facility level (1 in-charge of RCHs, and 2 RCH staff, who were traced at Mlandizi HC, and 1 RCHs staff having been traced at the Soga Dispensary). At community level, selection involved (2 CHWs, one linked with the Soga Dispensary and another one linked with the Mlandizi HC). These form the total sample size of 8 participants.

A multistage sampling approach was employed in the selection of both the wards and health facilities (HFs) where the targeted study participants could be traced. At first, two wards were randomly selected from the available 11 wards in the district, by ensuring inclusion of the totally rural and the semi-urban ones. Focus being on facilities owned by the government (popular as ‘public health facilities’), the next step was to identify facilities delivering RCHs. Randomisation was done by writing the names of the respective wards and dispensary on pieces of papers. The papers were folded and put into a box that was then shaken strongly for thorough mixing before allowing an independent person to pick the number of papers required to reveal the names of the selected study sample variable (e.g. a ward or dispensary).

The qualitative nature of the study was not oriented to presenting findings indicating statistical representativeness of the results, etc., only a depth understanding of what was considered as to whether it was important to have FP services involving men or not, how the services available were being utilised by men, modes of their delivery and the promoter or destructor factors for more policy relevant information and recommendations.

The RCH staff include those with professional skills for delivering the recommended services while CHWs were those dealing with the distribution of family planning commodities along (or concurrently) with other activities such as provision of health education and community sensitisation on various RCH related issues. The Mlandizi Health Centre was acting as a reference facility for some of the services that were not delivered at dispensary levels.

All study participants responded through answering face-to-face researcher-administered key informant interviews (KIIs) when approached individually. A working tool, namely, a KII guide was prepared in advance for this activity. The interviewers were flexible where it deemed necessary to fine-tune the questions in order to capture key information from the respondents concerned depending on their roles or positions in the health service delivery or management system and this was achieved through adoption of some probing techniques to maximise response or for additional clarity on a given study issue of interest.

### Data Collection Methods

Being aware that the goal of any qualitative interview is to maximise response by generating useful and sufficient data,^40^ an in-depth interview (IDI) Guide was used for the collection of data through key informant interviews (KIIs) sessions. The respective IDI Guide was developed after a reasonable review of an extensive literature on the subject. The review interest was in FP program environments within the health system that could encourage or discourage male involvement in FP in Tanzania. Lessons learned from past experience in Tanzania and the outside world, especially in Africa was traced in the reviewed articles to guide the design of the themes and subsequent study questions for use in IDIs.

With the consent obtained from the study individuals who were approached, all the interviews were tape recorded, conducted at the participants' respective health facilities and district office, and took between 40 and 60 minutes. All interviews were conducted in Kiswahili, the National language which is also fluently spoken by both the study investigator and the respondents. However, flexibility was allowed, as participants were informed that they could use even English if they felt it was more convenient at some points during the interview session. Each of the main questions asked was supported by several probes that came subsequently for clarity or validation on the answer(s) given for the preceding question. Kiswahili was a language commonly used by the respondents, some of whom could be heard using English terms such as ‘condoms’ instead of ‘mipira ya kiume’, ‘pills’ instead of ‘vidonge’, and ‘injection’ instead of ‘sindano’. Specifically, the main questions and their subsequent probes were directed to hearing stories from the respondents regarding the degree to which men (including adult and young ones) had a chance for being involved in modern FP program strategies, reasons for the observed current situation whereby questions were made on why the observed/reported to be facilitator/promoter factors or hindrances existed the way they seemed to be, to what extent or how the existing health care facilities and other FP service-related organisations provided a supportive environment for male involvement in modern FP service affairs, and finally, what could be done to increase modern FP service coverage to men.

### Data Handling and Analysis

A narrative analysis approach was primarily adopted in processing the data before reaching final interpretation. Each day after the interview was carried out, the research team could sit to debrief each other based on what they had experienced in the field regarding the attitudes and responses from their study participants. This was taken as an opportunity to enlighten and remind each other on certain pertinent or important issues for improving on as the interview process continued the next day. Summaries of key points garnered from the field and as recorded in the hand-written notes were prepared and preserved for informing or updating the next stages of the data analysis. Meanwhile, the tape-recorded interviews were spared a time for undergoing transcription verbatim and this was performed after the data collection exercise was completed.

The transcripts content was analysed in regards to the study theme. To arrive at this stage, transcripts had to undergo translation from Kiswahili language to English by ensuring that the original meaning as presented by the respondents were not distorted. A codebook was prepared, with key words or phrases. Such codes were then tagged in connection with text presented as seeming to represent particular meanings meant by the respondent responding to specific questions and as they seemed to correspond to the specific themes suggested for analysis. Thus, the respective thematic framework was used by a team of analysts and each at a time looked into the data independently, interpreted it accordingly, highlighted or identified the points that seemed recurrent and key, and with an eye on possible new and unique ones that could compare or contrast the rest as presented by either the same respondent or a different one, and brought them forward for a joint go-through, discussion and deliberations on what to take, what to leave and the way-forward. Inconsistencies in the details presented out of the transcripts were repeatedly examined, and where necessary, the exercise of listening to the record tapes for ascertaining statements and making corrections, if any, was repeated. Narratives were organised in such a way that statements seeming to carry similar meanings were categorised according to the way they were falling with the respective study theme.

Charts were drafted, with headings and sub-headings to capture the theme and sub-theme of interest for a compare and contrast exercise to allow triangulation of the results. The work of each analyst could be shared across interchangeably for reading and scrutinising so as to see how it informs or validates the work of another, and this helped to reduce individual analyst's bias and enhanced data validity.^46^ The final version of the transcripts translated in English was used for analysis.

Two major themes emerged and these addressed the (a) Extent of male involvement in RCHs specifically FP, reasons for the observed current situation on the degree of their involvement; and (b) Health providers’ views on structures and processes to support male involvement in RCHs. The latter theme had 3 sub-themes: (i) Organisation of FP services at health facility levels, focusing on service days and waiting time; (ii) Availability and adequacy of waiting and consultation rooms to accommodate couples as indicated by the respective room having at least 2 chairs, and (iii) Availability of all FP commodities applicable for the level of health facility.

Like all qualitative studies^47,48^, this stage involved going through the transcribed notes, one after another, tracing key points of interest and then comparing and contrasting the explanations/responses to particular questions or themes from different interviewees and finally reaching the decision on what to take or leave out, and what to take as general points and what to select as most important quotes for reporting.

### Ethics approval and consent to participate

The study was approved by the Research and Publications Committee of Muhimbili University of Health and Allied Sciences (MUHAS) with ethical clearance number MU/PGS/SAEC/Vol. XII/3. Permission to correct data was obtained from the district and respective health facilities administration. Participants were informed about the study and were told that their participation was voluntary. To maintain confidentiality, the information collected did not include names of participants.

## FINDINGS

### Demographic Characteristics of Respondents

As shown in Table 1, of the 8 KIs, 4 belonged to a cadre of registered nurse midwives, the rest were; a clinical officer, a public health nurse (PHN), and 2 trained community-based distributors of FP commodities (part of CHWs). The duration of their involvement in RCHs provision ranged between 2 and 30 years.

**TABLE 1:** Demographic Characteristic of Respondents

Characteristic	Number
Sex	
Female	6
Male	2
Cadre	
Registered nurse/midwives	4
Public health nurse	1
Clinical officer	1
Community health workers	2
Duration of their involvement in RCHs provision	
Less than 10 years	3
10-20 years	4
20 years and above	1

### KIs' views on Male Involvement in RCHs

All KIs were of the view that male involvement in RCHs issues is very important as it increases chances for uptake of various RCHs products and associated educational and counselling interventions. Some respondents emphasised the need for men to accompany their pregnant partners for HIV screening for PMTCT program based on which they can receive health education and counselling, especially in relation to FP and other family life aspects with a positive bearing on the health of all – the mother, the baby/child and the father, for instance issues of nutrition, proper use of prescribed medicines, and appropriate birth attendance for timely and safe pregnancy and childbirth. At one dispensary, a respondent expressed when pointing to what she called ‘reluctance of some men to attend Antenatal Care (ANC) clinic with their spouses in fear of being tested and found positive for HIV’. She remarked:

*There are still men who hesitate to accompany their spouses to antenatal clinics when asked by their spouses as we normally advise them to do. This requires more efforts to sensitise and persuade them since they fear from being tested and proven positive for HIV.*

KI, from Dispensary.

Other respondents reported increasing trend of male involvement in RCH issues. As argued, a number of men visit HFs with their partners, especially for pregnancy and child health services and HFs use the opportunity to test the couple for HIV under the PMTC program. Also, there are adolescent boys who visit HFs on their own for medical consultations, with particular interest in testing their status for HIV. The latter feedback was obtained from Mlandizi HC and Soga dispensary. In such cases, the health care workers utilise the opportunity to advise such clients on sexual and reproductive health services, with emphasis on cautioning them on how to prevent unplanned pregnancies by using condoms which also protects them and their partners from HIV infection. Despite the observed increase of males showing up for ANC and other RCHs, respondents noted that, previously it was not usual to see women coming with their male partners to the RCH service clinics due to the prevailing myth or perception that attendance for such services was a female affair. Below is a statement given by one informant whose opinion or experience resembled the feedback received from informants found at almost all HFs.

*“Initially it was strange to see a man accompanying his spouse, but now 5 to 6 men can be seen in this clinic per day and this makes counselling more meaningful especially when a joint decision is required”*

KI-1 from Health Centre.

Responding to a subsequent probe as to why there's observed increase of male involvement in RCHs including PMTC and FP services, the feedback obtained indicated that, the combined efforts to educate and sensitise the public, along with other practical interventions initiated by the government and its development partners (DPs) to support PMTC had positively contributed to the observed difference. DPs include private agencies and nongovernmental organisations (NGOs). These have worked hand in hand with the central government or the District Council to invest in money for extending consultation rooms and waiting lounges at HFs thus allowing accommodation of a larger number of clients at the same time. Therefore, the previous experience of clients having to wait for services outside the building and thus causing inconvenience could no longer be experienced. This comfort climate has convinced clients for RCHs including men. Moreover, respondents commended other efforts made by the district government authorities in cooperation with the District Council to support HFs to offer better quality of services. For example, giving men the priority, they seemed to desire for long time in relati on to their convincement to show up to access and use the recommended RCHs at the HF points of care that are closer to them.

These efforts included among other measures; the shortening of the clients' waiting time at the clinic by giving men first priority during consultations, strengthening community sensitisation meetings and using local community health representatives or CHWs as well as local government leaders through political meetings or local health committees as well as engaging religious leaders. These efforts were reported to be augmented or supported by the role played by mass media institutions that passed the message directly and widely to the public through various announcements, adverts and displays.

### Structures and Processes to Support Male Involvement in Reproductive Health Issues.

### Organisation of FP Services at Health Facility Levels

While 6 respondents claimed to have been providing health services in general and those of FP to have been delivered at almost all HFs throughout the week, one denied this experience by reporting that, there are some HFs in the district that do not deliver particular services on some days of the week for various reasons. One of the reasons reported was that, the respective service providers working at such facilities were designating some of the days each week specifically for delivering other reproductive health services particular to under-five children and pregnant women. This was testified by a respondent at a far remote based dispensary by commenting as follows:

“*We usually conduct FP clinics twice per week”*

KI from Dispensary

As clarified, the few personnel working at the respective HFs found themselves too occupied with a number of duties required for the mothers and their children, both during service delivery moments and the extra-service delivery. For example, the daily filling of information in the Health Management Information System (HMIS) – Mfumo wa Taarifa za Uendeshaji Huduma za Afya ‘MTUHA’ registers and cleanliness.

### Availability of Waiting and Consultation Rooms

Several informants commented further on the issue of ‘the clients’ waiting space' at the HF as one of the motivators for male's attendance for RCHs. The nature of the lounges where clients coming for services receive health education in groups, are also used as waiting place before being allowed to enter the medical or nursing consultation rooms or other places for receiving the required services. Thus, HFs that lack lounges force their clients to sit on wooden benches outside the clinic building while waiting for services. This is so inconveniencing during intense sunny or rainy days. Some of the HFs, particularly dispensaries, have no consultation rooms with capacity to accommodate all the clients attending especially when majority of such clients came in two's (as couples). This becomes a challenge on the part of frontline service providers who have to find ways for pleasingly serve such clients. One of the respondents had the following testimony:

*“Now, if a couple comes, I have to take a chair from another room or else I leave my seat for one of the clients and attend them while seated on the table or standing”*

KI-2 from Health centre.

Reports from respondents regarding waiting and service delivery space at HFs were somehow misleading. For example, respondents from either the same HF or a different one acknowledged what they reported ‘availability of consultation rooms that were sufficiently equipped with chairs for meeting the needs of the staff involved directly in the provision of services and at least accommodating one client at a time. Quickly, one could think that some of the HFs were well equipped in terms of sitting tools and physical space for service delivery purposes. However, since some clients attend as couples, the issue of space shortage remains. This is because a consultation room allowing only one client at a time would not suffice for clients attending as a couple. This brings about inconveniences not only to the clients but also to the service provider who would have to operate in an already meagre-spaced consultation room. Such scenarios prompt service provision frontline staff to attend to clients in a piecemeal fashion or rotation basis. This is not only inconveniencing, but also an inconsistent trait to the guidelines suggesting couple counselling for FP and PMTCT. As a coping mechanism, one of the options the respective service providers reported to have been taking was to schedule the services in such a way that different services are provided on different days of the week. Although this seems to be an effective solution on their side, it is inconveniencing on the side of the clients since clients as they have to wait for particular days to receive particular services despite the urgency.

### Waiting Time

Regarding the mechanisms used for coping with the inconvenience associated with long waiting time for services, respondents from all HFs pointed to the decision made to serve males first. It was claimed that, this decision was not made at the will and discretion of frontline service providers, but by the District Council's administration and health authorities in an effort to motivate men to show up for participating in RCHs. And as experience has shown, this move really encouraged men to participate in the recommended service programs such as FP, ANC in general and PMTCT in relation to HIV. On behalf of similar statements testifying what was attempted and achieved in relation to the latter experience, one informant made this remark:

*“We have shortened the waiting time for men who visit our facility for reproductive health services especially when they accompany their partners for PMTCT or bring their children to the under-five clinic by giving them priority for the services….. we give them services before others”*

KI-1 from Health Centre.

### Availability of FP Commodities

All respondents in this study reported their satisfaction with the availability/supply of FP commodities of all types, including condoms, pills, and Intrauterine Contraceptive Device (IUCD) most of the time at their workplace, although occasional stock outs of some of the commodities are sometimes experienced. In general, respondents noted that;

“*For the past month, Depo-Provera has been out of stock hence those using it are forced to use other methods affecting their compliance, leading to unplanned pregnancies. This brought about complaints directed to the providers as the cause of their unplanned pregnancies*”.

KI from dispensary

*“You see, I just have samples for demonstration purposes………. there are no Depo-Provera for the past month”*

KI-3, from Health Centre.

Respondents did not explicitly state reasons for the experienced commodity stock-outs and the researcher observed that respondents were adamant at speaking out the causes for the stock shortage. On further probe, respondents reported that stock shortages were due to delayed/late distribution or supply of the commodities from the district level, partly due to either transport arrangement interruptions or delayed supply from the central Medical Stores Department in Dar Es Salaam to the District Council. Furthermore, the researcher observed that all HFs shared the same experience and that due to increasing trend of RCH services utilisation, the district health department could not project or forecast the high demand for some of the products such as those of FP.

### KIs' Views on how to Improve Services to Support and encourage Men's Involvement in Family Planning Issue

Six KIs' responses, on how to make responsive FP delivery system were found to be concentrated around addressing issues mainly relating to increasing the number and sizes of consultation rooms so that they can accommodate couples showing up for health education and other services. This is so important since lack/shortage of such rooms made HFs especially at dispensary, provide services in alternate days since available space could not accommodate all RCH clients at ago. This is an inconvenience not only to the service providers, but also to the clients who miss getting all the essential services they could have received in a single day visit to RCHs. To address this issue one respondent was of the opinion that efforts or plans are made and translated into reality by ensuring that every RCHs clinic has a waiting lounge containing a television monitor. Through this facility, clients could follow the service providers' teachings complemented with watching the demonstrations displayed on the screen with health information relating to male involvement in RCHs services.

## DISCUSSION AND POLICY RELEVANCE

The present study collected useful information, reflecting the status of male involvement in RCHs and the importance of availability of well-equipped health care infrastructure and essential commodities for provision of standard FP services to the needy population in a rural district setting. It is obvious from the reports presented by KIs that Shortage of consultation rooms is a discouragement to RCH utilisation.

Apart from undermining the client's privacy during consultation moments, it also leads to disappointments to clients due to delays in seeking for services/care at the HFs. Also, disappointments can rise in a situation where clients seek for services on a particular day and yet the HFs' number of appointments are already above their capacity of personnel to attend to them within a short time, thus causing long queues at the health facility.

These limitations are consistent with what is widely documented by other researchers in other parts of Tanzania^7^ and elsewhere in Africa.^49^ Access limitations due to long waiting time brought about by HF understaffing as well lack of waiting lounges/rooms at HF level was also reported by pregnant women attending ANC services in Mkuranga and Mufindi districts, Tanzania.^50,51^ Dysfunctional or poorly equipped HFs has also been reported to be barriers to uptake of FP and other sexual and reproductive services in many other countries, within and outside of Africa.^52^ To address this limitation, governments in collaboration with bilateral and multilateral agencies continuously invest more in structures, equipment, supplies and human resources.^25^

Respondents noted that male involvement in RCHs improved or enhanced the uptake of various reproductive, maternal and newborn interventions such as PMTCT and FP, as well as services delivered in programs that deal with parental engagement in childbirth and child care matters. Such improvement is supported by a number of programs run by the government in cooperation with, or exclusively run by NGOs.^55,56^ This observation was due to the fact that key staff responsible for services delivery received long and short-term training on various RCH aspects which could be the reason for their positive attitude towards the campaign for involving men in the respective service programs or initiatives. In a similar way, reports from studies made in many other developing countries show that most health workers recognise and appreciate male involvement in maternal, newborn, child, adolescent, youth and adult health programs and this has in one way or another contributed to their increased motivation for delivering such services and resulting into increased male attendances.^4–6,57^ Thus, health workers' recognition of the importance of male involvement in RCHs is a good indicator of positive attitude towards various interventions aimed at promoting male involvement in RCHs. These findings, therefore, suggest a need for increasing knowledge and sensitisation of health providers to increase their responsiveness in encouraging men to engage in reproductive health services.

Key Informants reported an upward trend of men visiting health facilities for various RCHs including accompanying their spouses for HIV screening to PMTCT and family planning centres. This observation might imply that the campaigns and associated interventions made so far in the district have enhanced men's awareness and knowledge about the issue. However, these strategies have not been fully investigated on, and therefore, further studies are recommended in order to verify observed results elsewhere in Tanzania.^58,59^ Evidence from other studies carried out in developing countries demonstrates that, when couples and communities at large possess high health literacy, staying in an environment with proper service working infrastructures and with availability of essential supplies, such couples are likely to be motivated to seek for recommended RCH services in a timely and correct manner.^52^ Also, such couples have been observed to have increased informed decision making, ownership, and responsibility which inturn lead to increased participation in PMTCT aginst HIV.^60^

There is still low male involvement in FP issues and this is expected to have negative implications on the reliability and sustainability of the apparent rise in the trend of their involvement in the study district. Thus, despite the reported observance on the increase in male involvement in RCHs in Kibaha district coupled with the high service providers' recognition of the importance of such involvement, the identified challenges might discourage potential clients to show up for the services and thus, the general public may lose trust in the existing health service system.^61^ This is supported by evidence from studies carried out in other developing countries, reporting low male involvement in FP issues due to systemic/institutional, behavioural, financial and geographical factors. Systemic/institutional factors include; health facility building and infrastructure status, availability of essential supplies or commodities, number, skills and motivation of service providers.^4,62^ Behavioural factors are attitudinal in nature and these are rooted from cultural limitations.^14,63^ Financial factors are economic cost related and geographical factors are those associated with long travel distance and waiting time.^7,11,25,62,64,65^

### Study Limitations

The present study, however informative it is on FP service utilisation by men in Kibaha district, the study is subject to a number of shortcomings, namely; time and financial constraints, thus, the sample size considered was very small. A sample of 8 participants raises questions relating to data saturation and representation, even though qualitative studies do not necessarily require considering large samples when compared to statistics oriented studies, observations of this study need to be tested for validity and reliability by considering a larger sample size.^48,61^ The unwillingness of some respondents to disclose confidential information to the researchers was also a possible limitation to this study. There seemed a point when the participants were hesitant to disclose the reasons for the observed suboptimal coverage of men in FP and other RCHs that could be attributed to systemic deficiencies. Respondent's and Researchers' biases due to personal view or limited scope/level of understanding of the issues under this study was also a possible limitation. Different studies have noted that the place of interview/data collection, researcher/investigator's bias, and interviewee' expectations are matter of concern since they contribute to influencing the responses from the study participants approached.^66^ There was also limited focus since this study handled aspects relating to FP and RCHs differently. Most of these limitations could not be addressed given the available resources of time, human and financial nature.

## CONCLUSIONS

There is no question about the role men can play if involved in FP, maternal, child and other reproductive health issues as highly recognised by the service providers interviewed in this study. Nevertheless, it suffices to appreciate that more efforts are needed to increase and sustain their involvement for betterment. The observed increase in the tendency of males to accompany their partners to RCHs clinic should be nurtured through deliberate and concerted efforts. This would involve supporting policy and guidelines for implementing the - strategies recommended, involving of key stakeholders both at institutional and family level, and ensuring that there is sufficient supply of essential products and well-equipped health infrastructure and staffing systems, supplemented with periodic evaluation and analysis of achievements and milestones to enhance and sustain male involvement/participation.
